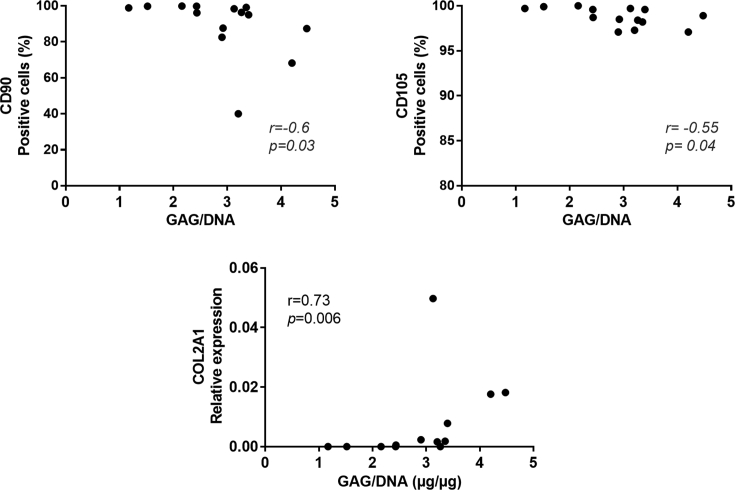# The synovial fluid from patients with focal cartilage defects contains mesenchymal stem/stromal cells and macrophages with pro- and anti-inflammatory phenotypes

**DOI:** 10.1016/j.ocarto.2020.100039

**Published:** 2020-02-19

**Authors:** John Garcia, Charlotte Hulme, Claire Mennan, Sally Roberts, Yvonne M. Bastiaansen-Jenniskens, Gerjo J.V.M. van Osch, Bernhard Tins, Peter Gallacher, Karina Wright

**Affiliations:** a School of Pharmacy and Bioengineering, Keele University, Keele, Staffordshire, ST5 5BG, UK; b The Robert Jones and Agnes Hunt Orthopaedic Hospital NHS Foundation Trust, Oswestry, Shropshire, SY10 7AG, UK; c Department of Orthopaedics, Erasmus MC University Medical Center, 3000 CA Rotterdam, Netherlands

**Keywords:** Synovial fluid, Osteoarthritis, Macrophages, Inflammation, Mesenchymal, Stromal/stem cells

## Abstract

**Objective:**

The synovial fluid (SF) of patients with focal cartilage defects contains a population of poorly characterised cells that could have pathophysiological implications in early osteoarthritis and joint tissue repair. We have examined the cells within SF of such joints by determining their chondrogenic capacity following culture expansion and establishing the phenotypes of the macrophage subsets in non-cultured cells.

**Design:**

Knee SF cells were obtained from 21 patients receiving cell therapy to treat a focal cartilage defect. Cell surface immunoprofiling for stem cell and putative chondrogenic markers, and the expression analysis of key chondrogenic and hypertrophic genes were conducted on culture-expanded SF cells prior to chondrogenesis. Flow cytometry was also used to determine the macrophage subsets in freshly isolated SF cells.

**Results:**

Immunoprofiling revealed positivity for the monocyte/macrophage marker (CD14), the haematopoietic/endothelial cell marker (CD34) and mesenchymal stem/stromal cell markers (CD73, CD90, CD105) on culture expanded cells. We found strong correlations between the presence of CD14 and the vascular cell adhesion marker, CD106 (r = 0.81, p = 0.003). Collagen type II expression after culture expansion positively correlated with GAG production (r = 0.73, p = 0.006), whereas CD90 (r = −0.6, p = 0.03) and CD105 (r = −0.55, p = 0.04) immunopositivity were inversely related to GAG production. Freshly isolated SF cells were positive for both pro- (CD86) and anti-inflammatory markers (CD163 and CD206).

**Conclusions:**

The cellular content of the SF from patients with focal cartilage injuries is comprised of a heterogeneous population of reparative and inflammatory cells. Additional investigations are needed to understand the role played by these cells in the attempted repair and inflammatory process in diseased joints.

## 1 Introduction

A growing body of research has demonstrated that joint degeneration in osteoarthritis (OA) is not only due to mechanical attrition (through progressive wear or trauma) but is also facilitated, at least in part, by an inflammatory environment [1,2]. Focal defects of the cartilage in the knee are associated with further degradation of cartilage and are a risk factor for OA [3]; however the cellular events involved in this process are still poorly understood. The synovial fluid (SF), which contains both its own population of cells and molecules produced by other joint tissues, provides a means of evaluating the inflammatory status of the local environment. Whilst molecules such as cytokines have been extensively explored in the SF as potential biomarkers of OA [4], few studies have investigated the cellular content of the SF, particularly in early OA [5].

Previous reports revealed the presence of immune cells, such as macrophages, lymphocytes and natural killer cells, in the SF of non-rheumatoid, end-stage OA patients [6,7]. These cells form part of the poorly understood innate immune system of the joint, that could hold important information on OA pathophysiology, as well as therapeutic targets at an early stage of disease progress and may contribute to disease pathophysiology. The changes that occur in early OA affect the articular cartilage (increased catabolic enzyme activity), subchondral bone (subchondral bone remodelling and osteophyte formation), meniscus (fibrillation of avascular region), synovium (hyperplasia and lymphocyte infiltrates), tendons and menisci (ruptures and tears) [8].

A population of mesenchymal stem/stromal cells (MSCs) with chondrogenic potential has also been characterised from the knee SF (SF-MSCs) [9] of early OA patients and their prevalence increases in the end stages of the disease [10]. The biological properties and role of these SF-MSCs in the early stages of OA development or attempted joint repair is still unclear. The SF may also contain chondrocyte populations originating from cartilage fragments in a damaged articular joint. In the present investigation, we firstly analysed the characteristics of plastic adherent SF cells in culture from early OA patients for markers of chondrogenesis, stem cells and immune cells. Secondly, the unexpected presence of CD14 on the culture-expanded cells prompted us to determine the phenotype of these monocyte/macrophages in freshly isolated, non-cultured, SF cells from patients receiving autologous cell implantation (ACI), a procedure recommended for patients with focal cartilage defects but not end-stage OA.

## 2 Methods

### 2.1 Patients

Ethical approval was given by the National Research Ethics Service (11/NW/0875) and all patients provided written informed consent before SF was taken. Approximately 3–20 mL of SF were obtained via arthrocentesis, as previously described [10], from the knees of 21 patients (13 males, 8 females, mean age = 35 ± 11 years) undergoing ACI to treat focal cartilage defects assessed arthroscopically and using magnetic resonance imaging (MRI) (Table 1). ACI involves a cartilage harvest from a minor load bearing region of the joint (stage 1) and the implantation of cells culture expanded from the biopsy, into the joint 2–3 weeks later (stage 2). Patients included in this study had a focal cartilage defect, while patients with end stage OA and a Kellgren-Lawrence (KL) score above 2 were excluded from this study (average KL score = 1.1 ± 0.9).

**Table 1:** Demographics of patients from which SF cells was obtained. Only non-cultured cells were used for macrophage characterisation.

Patient number	Gender	Age	BMI	Defect size (mm^2^)	KL Score	Culture expansion?
1	Male	22	35.0	638	2	Yes
2	Female	48	29.5	200	2	Yes
3	Male	21	37.6	225	0	Yes
4	Female	19	34.1	400	2	Yes
5	Male	36	23.6	450	2	Yes
6	Male	28	30.5	150	1	Yes
7	Male	28	27.0	105	2	Yes
8	Female	30	27.8	32	1	Yes
9	Male	47	26.6	250	0	Yes
10	Female	35	23.8	200	0	Yes
11	Male	29	19.4	110	2	Yes
12	Male	30	36.3	100	0	Yes
13	Female	36	21.1	150	0	Yes
14	Female	63	23.7	400	2	Yes
15	Female	42	25.0	360	2	Yes
16	Male	37	37.9	300	0	No
17	Male	27	28.0	250	1	No
18	Male	52	22.8	300	0	No
19	Male	27	24.8	500	2	No
20	Male	40	28.0	750	1	No
21	Female	46	34.0	300	1	No

### 2.2 Isolation of SF cells

The SFs were centrifuged at 800*g* for 15 min immediately after surgical intervention and no evidence of heavy blood staining was found in the samples. The supernatant was removed and the resulting pellet was either resuspended in complete culture medium (n = 15) comprised of Dulbecco's Modified Eagle's Medium/F-12 (DMEM/F-12, Life Technologies) with 1% (v/v) penicillin/streptomycin (P/S, Life Technologies) and 10% (v/v) foetal bovine serum (FBS, Life Technologies) for monolayer expansion, or prepared directly for flow cytometry.

### 2.3 Multichromatic flow cytometry

Table 2 provides details of the clones, isotypes and fluorochromes of all the antibodies used for flow cytometry in this study. A minimum of 10 000 cells were acquired and analysed for each marker. Cultured cells were expanded by seeding at 5000 cells/cm^2^ and trypsinisation up to passage 3, after which multichromatic flow cytometry using fluorochrome-conjugated antibodies (all BD Biosciences) was used to determine the positivity of certain immune cell markers (CD14-PercP-Cy5.5, CD19-BV421, CD45-PE), the vascular cell adhesion marker (CD106-APC), haematopoietic/endothelial/adipose cell marker (CD34-APC), the major histocompatibility complex class II marker (HLA-DR-APC), the MSC markers (CD90-PE, CD73-BV421 and CD105-APC), and putative chondropotency markers (CD39-APC, CD44-PercP-Cy5.5 [11], CD49c-PE [11], CD151-PE [11], CD166-BV421 [12], and CD271-BV421 [13]), were also examined as we described previously [14].

**Table 2:** Fluorochrome conjugated antibodies used for multichromatic flow cytometry.

Marker	Antibody Clone	Isotype control	Fluorochrome
**ISCT**^a^**MSC markers**			
CD73	AD2	Mouse IgG1	BV421
CD90	5E10	Mouse IgG1	PE
CD105	266	Mouse IgG1	APC
CD14	MϕP9	Mouse IgG2b	PercP-Cy5.5
CD19	HIB19	Mouse IgG1	BV421
CD34	581	Mouse IgG1	APC
CD45	HI30	Mouse IgG1	PE
HLA-DR	TU36	Mouse IgG2b	APC
**Chondropotency markers**			
CD44	G44-26	Mouse IgG2b	PercP-Cy5.5
CD166	3A6	Mouse IgG1	BV421
CD49c	C3 II.1	Mouse IgG1	PE
CD106	51-10C9	Mouse IgG1	APC
CD151	14A2.H1	Mouse IgG1	PE
CD39	TU66	Mouse IgG2b	APC
CD271	C40-1457	Mouse IgG1	BV421
**Macrophage markers**			
CD14 (pan marker)	M5E2	Mouse IgG2a	APC
CD80 (M1)	L307.4	Mouse IgG1	PE
CD86 (M1)	2331 (FUN-1)	Mouse IgG1	FITC
CD206 (M2)	19.2	Mouse IgG1	BV421
CD163 (M2)	GHI/61	Mouse IgG1	PerCP-Cy5.5

a ISCT: International Society for Cellular Therapy.

To explore the various subsets of macrophages in freshly isolated SF cells from arthrocentesis (n = 6) at baseline (prior to stage 1 of ACI, which is the time of cartilage harvest) and 2–3 weeks later (prior to stage 2, which is the time of cell implantation), fluorochrome-conjugated antibodies against the M2 markers CD206-BV421 and CD163- PerCP-Cy5.5 [15,16], and M1 markers CD80-PE and CD86-FITC [17,18], were used together with the pan macrophage marker CD14-APC in multichromatic flow cytometry. After immunostaining, the cells were incubated with a haemolysis buffer (BD Biosciences) for 1 min to remove any red blood cells. Matching fluorochrome-conjugated isotype controls were used for each antibody (all BD Biosciences). For all flow cytometry analyses, gating was first established (at 1% positivity) on the isotype controls and then applied to the corresponding markers. Stage 1 and 2 cell samples were analysed separately (including isotype controls). CD14^+^ cells were first gated as part of the total cell population. The M1 and M2 positive cells were gated individually and as double positive combinations within the CD14^+^ cells. All gate cut-offs were established based on isotype controls (1%).

### 2.4 RNA extraction and Quantitative Real-Time polymerase chain reaction (qRT-PCR)

The expression of chondrogenic genes Sex-Determining Region Y-Box 9 Protein (*SOX9*), aggrecan (*ACAN*), collagen type II (*COL2A1*) and frizzled-related protein 1 (*FRZB*) and hypertrophy genes collagen type X (*COL10A1*) and activin receptor-like kinase 1 (*ALK1*) [19], were assessed by qRT-PCR at passage 3. Briefly, messenger RNA was isolated from cultured cells using an RNeasy kit (Qiagen) and cDNA was generated using the High-Capacity cDNA Reverse Transcriptase Kit (Applied Biosystems), according to manufacturer's recommendations. qRT-PCR analysis was conducted on a Quant Studio 3 Quantitative Real-Time PCR System (Applied Biosystems) using SYBR green QuantiTect primer assays for *SOX9, ACAN, COL2A1, FRZB, COL10A1, ALK1* (Qiagen). Relative gene expression was determined by the comparative C_t_ method (2 ^−ΔCt^), using the reference genes peptidylprolyl Isomerase A (*PPIA*) and TATA-box binding protein (*TBP*). These reference genes were the two most stable from a screening test of five genes (data not shown).

### 2.5 Chondrogenic differentiation and histological analysis

At passage 3, SF cells were induced towards chondrogenic differentiation for 28 days in 3D pellet culture by centrifuging (8 mins at 350*g*) 2 × 10^5^ cells and culturing the resulting pellet in chondrogenic medium which consisted of complete culture medium supplemented with Insulin-Transferrin-Selenium (1% v/v, Life Technologies), ascorbic-acid (0.1 mM, Sigma), dexamethasone (10 nM), sodium pyruvate (1 mM), linoleic acid (20 μM) and Transforming Growth Factor-β1 (10 ng/ml, PeproTech). Chondrogenic differentiation was assessed by scoring of toluidine blue-stained cryosections (0–9, with 9 being the highest levels [20]) and GAG quantitation using the dimethylmethylene blue assay (DMMB). Spearman's correlation analyses of gene expression after culture expansion and the positivity of surface markers and the production of GAGs in the pellets were performed.

### 2.6 Statistical analysis

All statistical analyses were conducted in GraphPad Prism version 7. The Shapiro-Wilk normality test revealed that the data for flow cytometry, gene expression, GAG/DNA and histology scores were not normally distributed, and as a result, non-parametric Spearman's correlations were used for determining relationships between immunopositivity or gene expression and the production of GAGs in the pellets. Two-way ANOVAs were used to determine the difference in means between stage 1 and stage 2 macrophage positivity in SF cells. Data, unless otherwise stated, are shown as median with the interquartile range (IR), and *p*-values less than 0.05 were considered significant.

## 3 Results

### 3.1 Immunoprofile of culture expanded SF cells in early OA patients

Cultured SF cells displayed high median positivity for the MSC markers CD73 (97.4%, IR 43.1–99.3), CD90 (97.4%, IR 40.0–99.1), CD105 (98.9%, IR 98.2–99.7) and the immunomodulatory marker CD106 (91.2%, IR 77.4–96.3). Although CD45, CD19 and HLA-DR were absent (<1%), CD14 (53.0%±, IR 19.0–84.3) and CD34 (23.3%, IR 6.4–35.8) were present with significant patient variation (Fig. 1A). Strong correlations were found between the presence of CD14 and CD34 (r = 0.7, p = 0.03), and between CD14 and CD106 (r = 0.81, p = 0.003) on cultured cells (FigS1 Supplementary Data 1). The chondrogenic markers CD44 (99.7%, IR 98.2–99.9) and CD151 (99.9%, IR 99.1–99.8) were positive on all of the culture expanded SF cells from all patients investigated, whilst CD39 (21.4%, IR 16.7–28.2), CD49c (18.6%, IR 13.6–40.7) and CD166 (17.6, IR 6.1–32.6) showed varying levels of immunopositivity (Fig. 1B). Cells showed low levels of immunopositivity for CD271 (3.97, IR 2.9–9.0).

**Fig. 1:**
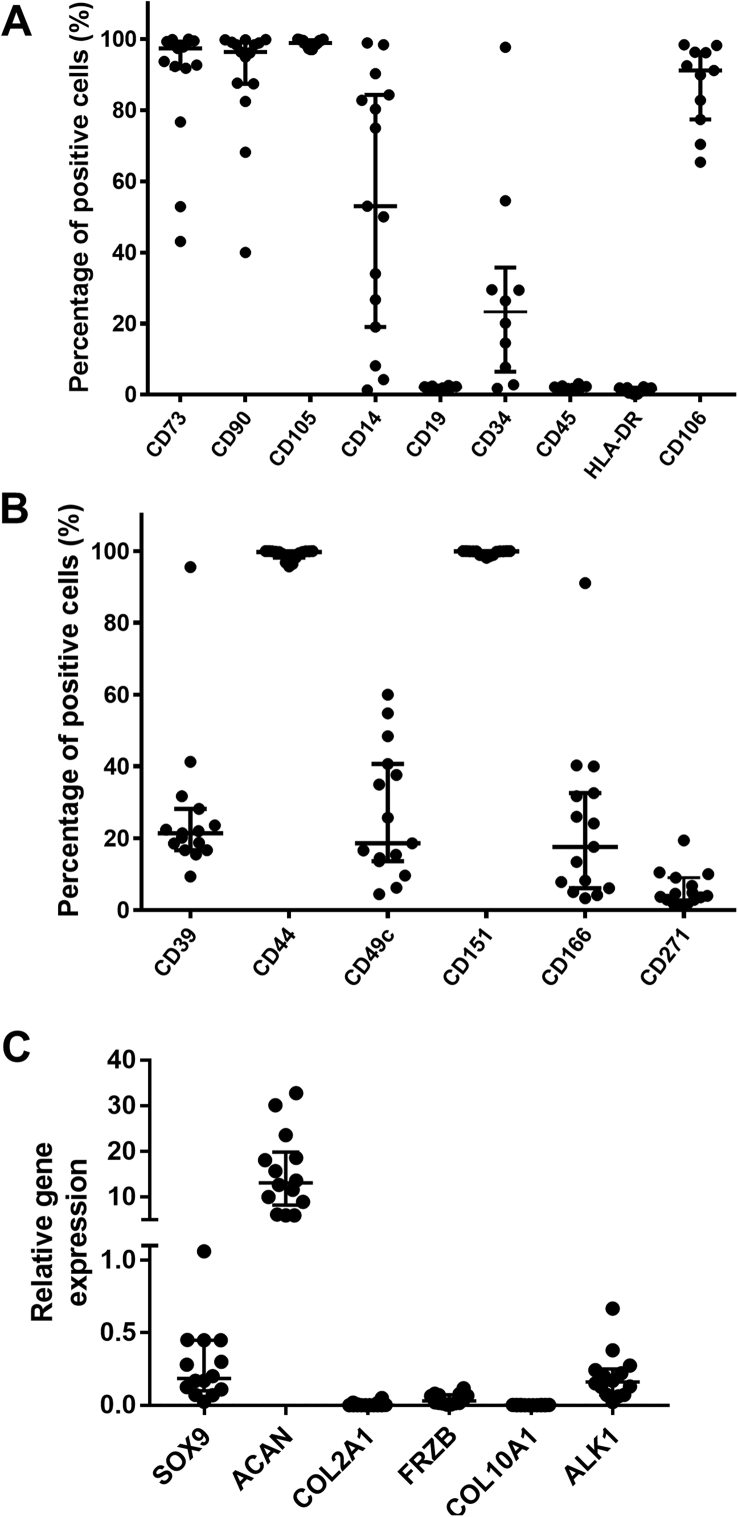
A) Flow cytometry was used to determine the MSC cell surface immunoprofile of SF cells (P3), before chondrogenic induction. This panel consists of MSC markers (CD73, CD90, and CD105, n = 15) as well as immune cell markers (CD14, CD106, CD19, CD45 and HLA-DR, n = 10) and one haematopoietic marker (CD34, n = 10). (B) Flow cytometry was also used to determine the percentage of SF cells that were positive for surface markers known to be indicative of chondrogenic potency. (C) The expression of chondrogenic (SOX9, ACAN, COL2, and FRZB) and hypertrophic (COL10A1 and ALK1) genetic markers was determine by qRT-PCR, with PPIA and TBP used as reference genes (n = 14). Scatter plots show medians with interquartile ranges, with each dot representing a patient.

### 3.2 The chondrogenic capacity of SF cells is variable in early OA patients

**Fig. 2:**
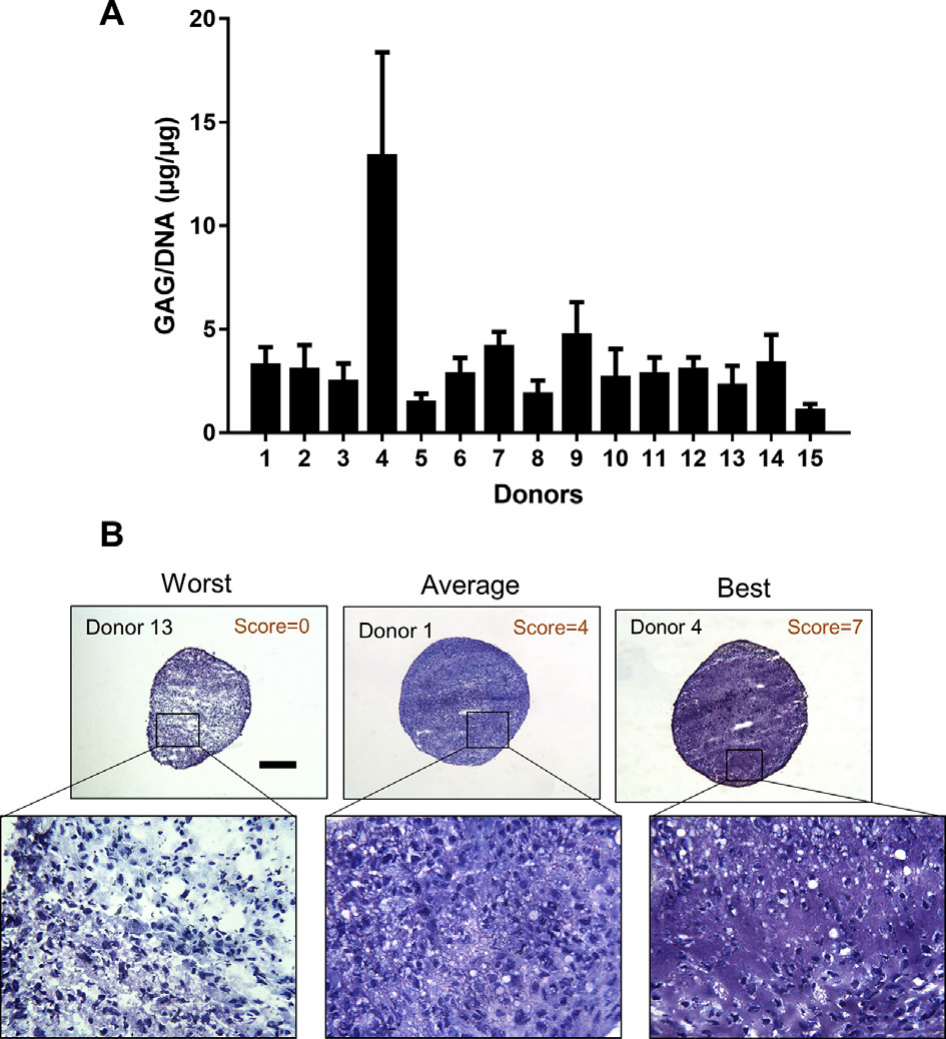
A) Production of GAGs by SF cells. The GAGs from chondrogenic pellets of SF cells were quantified using the DMMB/DNA assay (n = 15). Error bars represent the standard deviation of triplicate pellets for the same patient. (B) Representative images of toluidine blue stained sections of SF cell pellets with histology scores (n = 10). The images indicate the worst (left), average (central) and best (right) chondrogenic differentiation in the samples studied using the Bern scoring system that ranges from 0 to 9 (0 = poor chondrogenesis, 9 = good chondrogenesis). Scale bar = 200 μm.

**Fig. 3:**
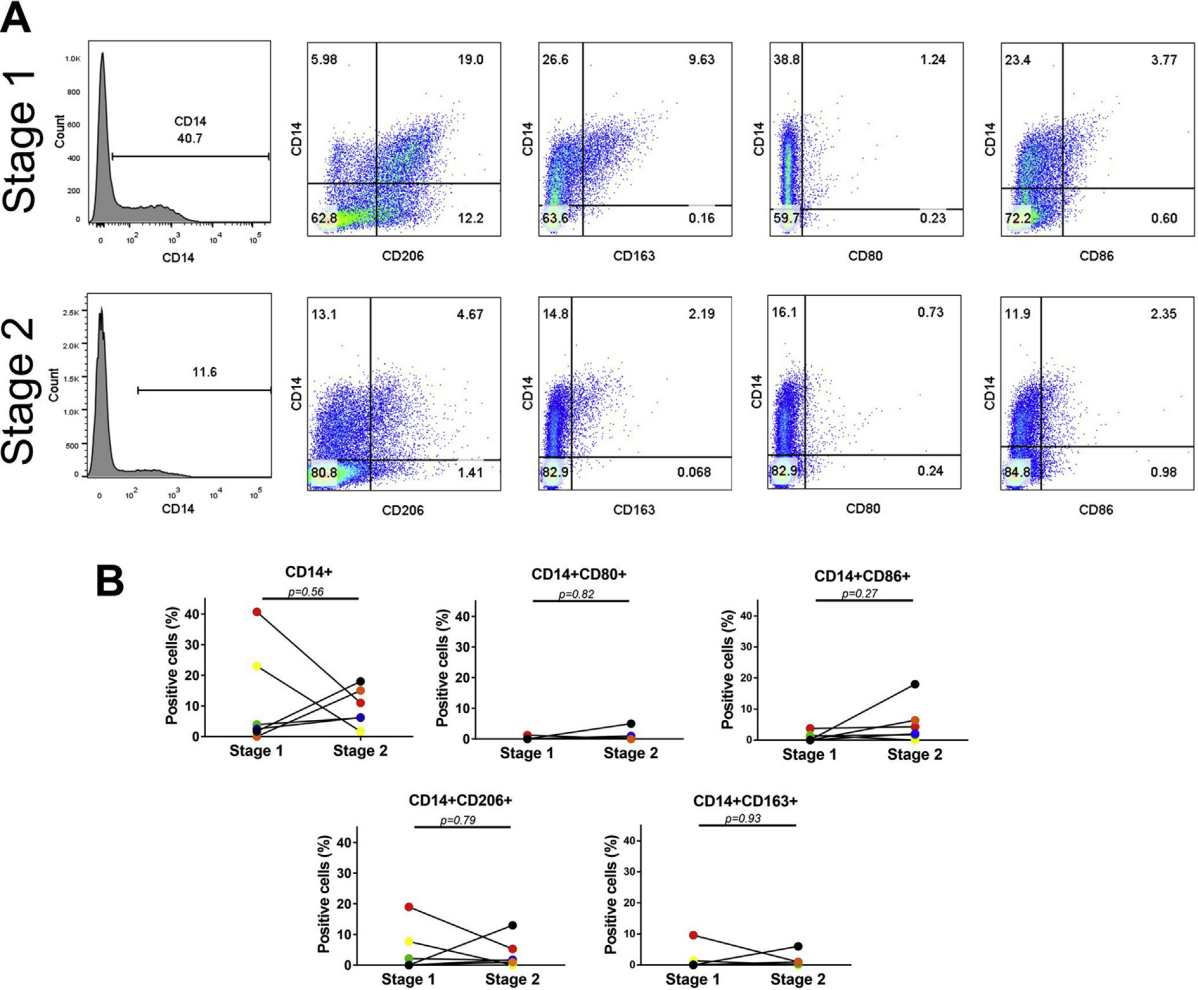
A) Representative scatterplots and histograms of macrophage analysis in fresh SF from stage 1 and stage 2 of patient 16 receiving cell therapy. Multicolour flow cytometry was used to identify and quantify macrophage subsets using antibodies against CD14 (pan macrophage marker), CD80, CD86 (both M1), CD206 and CD163 (both M2). Gates were established from isotype controls and values in the quadrants represent the percentage of positive cells. (B) Comparison of the macrophage profiles in matched stage 1 and stage 2 SF samples from patients 16–21 (n = 6).

SF cells showed considerable variability between patients with regards to the expression of chondrogenic and hypertrophic genes before differentiation. The markers *SOX9*, *ACAN*, and *ALK1* were highly expressed, while *COL2A1*, *FRZB* and *COL10A1* showed minimal or no expression (Fig. 1C). After chondrogenic induction, SF cells produced a median of 3.1  μg (IR 2.4–3.4) of GAG/μg of DNA (Fig. 1, Fig. 2, Fig. 3A). Histology indicated variable toluidine blue staining, both between samples and sometimes within a pellet (Fig. 2B). The strongest toluidine blue staining was seen in pellets formed from patient 4 which produced 12.7  μg GAG/μg of DNA (SD ± 4.9), which was supported by the histological analyses. Thus, the mean GAG/DNA results for patient 4 created an outlier that was 3.4 standard deviations above the mean of all of the samples.

### 3.3 Correlation analyses between chondrogenic markers and chondrogenic differentiation

None of the putative chondrogenic surface markers correlated significantly with the production of GAG by SF cells (Table 3). Due to the unexpected variability in positivity of CD14, CD73 and CD90 in expanded SF cells across patients, analyses were run to determine whether the positivity of these markers was associated with the production of GAG by the cells, but no significant correlations were found. When the outlier of patient 4 was excluded from the analyses, CD90 (r = −0.6, *p* = 0.03) and CD105 (r = −0.55, *p* = 0.04) had a moderate negative correlation with the production of GAG/DNA (Table 3, scatterplots found in FigS2 Supplementary Data 2). Similarly, none of the genetic markers examined correlated with the production of GAG (DMMB or histology scores) in SF cells after chondrogenic induction, but when the outlier of patient 4 was excluded, GAG/DNA production strongly correlates with *COL2A1* expression (r = 0.73, p = 0.006) (Table 3). Interestingly, the cells from patient 4, which produced the highest amount of GAG, also showed the highest expression of *SOX9*.

**Table 3:** Spearman correlation coefficient values for the correlation between chondrogenic outcome (GAG/DNA and histology scores) and baseline expression of genetic and surface markers.

	GAG/DNA (n = 15)		Histology score (n = 9)	
	r	p values	r	p values
**Genetic markers**				
*SOX9*	0.02	0.98	0.41	0.31
*COL2A1*	0.50	0.07^a^	−0.48	0.23
*ACAN*	0.33	0.25	−0.22	0.62
*FRZB*	−0.01	0.97	0.29	0.50
*COL10A1*	0.17	0.55	−0.57	0.14
*ALK1*	0.07	0.8	−0.29	0.48
**Surface marker**				
CD49c	−0.20	0.40	−0.04	0.90
CD166	0.043	0.88	−0.18	0.64
CD39	−0.08	0.79	−0.25	0.52
CD271	0.44	0.11	0.08	0.85
CD73	−0.03	0.91	−0.14	0.71
CD90	−0.51	0.06^a^	−0.11	0.78
CD105	−0.28	0.30^a^	−0.16	0.69
CD14	−0.43	0.11	−0.38	0.32
CD34	−0.18	0.63	0.003	0.99

a When the outlier of patient 4 was excluded from analyses, correlations were significant as follows: COL2A1 (r = 0.7, p = 0.006); CD90 (r = −0.6, p = 0.03); CD105 (r = −0.55, p = 0.04).

### 3.4 Pro-inflammatory and anti-inflammatory monocyte/macrophages are a subset of non-cultured SF cells

**Fig. 4:**
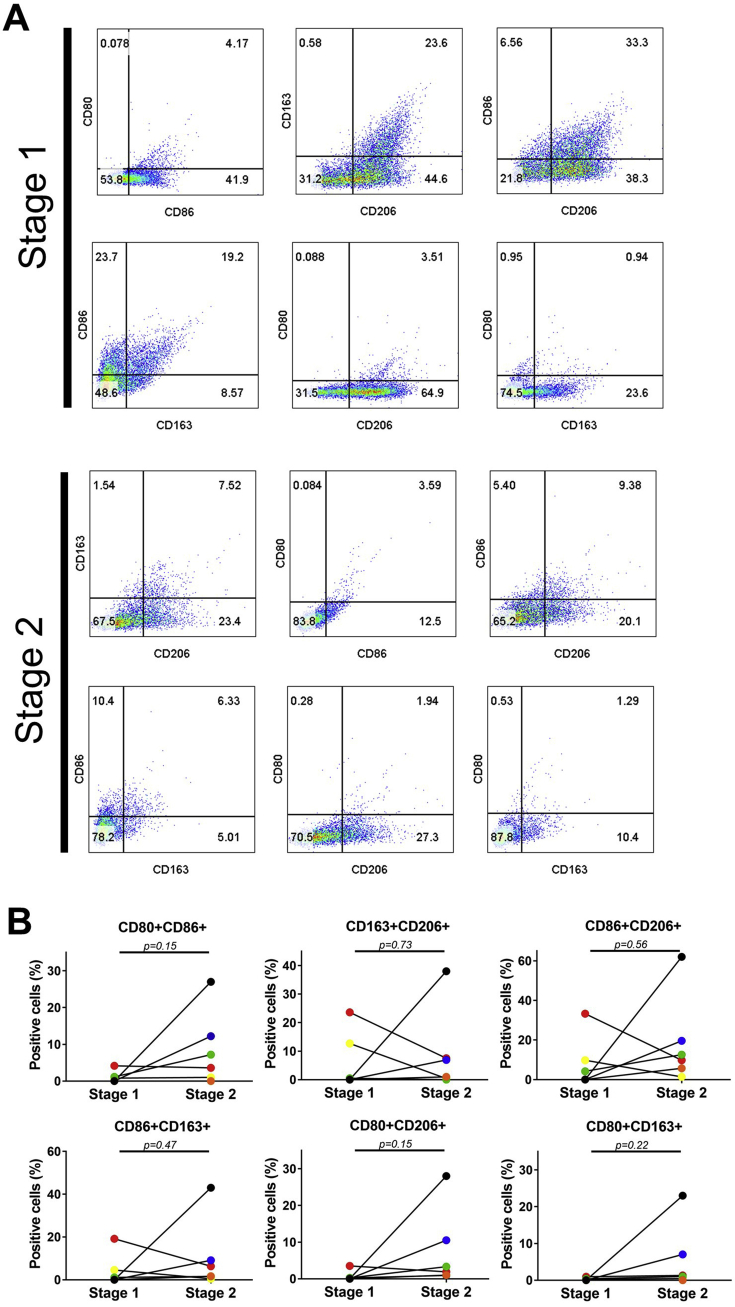
A) Representative scatterplots of the co-positivity of M1 and M2 markers on macrophages in the SF of patient 16 at stage 1 and at stage 2. Cells that were positive for CD14 were gated and used to plot the co-positivity of M1 and M2 markers. Gates were established from isotype controls and values in the quadrants represent the percentage of positive cells. (B) Comparison of the M1/M2 co-positivity profiles in matched stage 1 and stage 2 SF samples from patients 16–21 (n = 6).

Multichromatic flow cytometry confirmed the presence of CD14^+^ cells (3.2% of total SF cells, IR 1.3-27.4) at baseline (stage 1) and post-cartilage harvest (stage 2) (8.6%, IR 5.0–15.8). For the M1 markers, we found virtually no CD14+/CD80+ (0.0%, IR 0.0-0.31) or CD14+/CD86+ (0.6%, IR 0-2) co-positive cells at stage 1, with a marginal increase in CD14+/CD86+ (3.1%, IR 1.28-3) at stage 2 (Fig. 3A and B). Cells that were CD14+/CD206+ and CD14+/CD163+ co-positive were detected at both stage 1 (1.0, IR 0-10.5 and 1.0%, IR 0-3.4 respectively) and at stage 2 (1.7%, IR 0.75-7.2 and 1.5%, IR 0.2-2.3 respectively). No statistical difference between the two stages was found for any of the markers tested. Further analyses confirmed there to be a subpopulation of monocyte/macrophages containing both M1 markers, i.e. CD14+/CD80+/CD86+ (Fig. 4). Similarly, co-positivity for M2 markers, i.e. CD14+/CD206+/CD163+ was also observed. Interestingly, some CD14^+^ SF cell samples were simultaneously positive for the M1 marker CD86 and either CD206 or CD163. Whilst CD14+/CD80+/CD206+ and CD14+/CD80+/CD163+ cells were not seen at stage 1, a small increase at stage 2 was detected.

## 4 Discussion

The aim of this study was to characterise cells found in the SF of patients at an early stage of OA, categorised as having focal cartilage injuries, immediately following arthrocentesis and in the plastic-adherent sub-cultured population. Our findings suggest that the SF of these patients contains a heterogeneous population of cells. The positivity of CD14 could indicate the presence of monocytes/macrophage-like cells, possibly originating from the synovium, infrapatellar fat pad and/or subchondral bone in the event of deep cartilage defects. The detection of CD34 also suggests the presence of haematopoietic cells, endothelial cells or adipose derived mesenchymal stem cells [21]. The absence of CD19 confirms that lymphocytes (B cells) did not persist in the culture of SF cells [22], whilst the lack of any HLA-DR positivity suggests a low state of activation for any monocytes/macrophages present in the SF cultures. The surface marker CD106 is involved in the recruitment of immune cells from blood vessels [23], but also identifies a subpopulation of MSCs with enhanced immunomodulatory properties [24]. High positivity for CD106, together with its correlation with CD14, could explain the presence of macrophage-like cells in the SF, i.e. since an increase in CD106 could engender more infiltration of immune cells into the joint. The frequency of macrophages in the synovium of the knee joint is known to be augmented in both the early and late phases of OA, which contributes to the inflammatory state of the joint [25,26]. Another study assessing the cell surface epitopes of SF cells after acute knee ligament injury reported a similar lack of lymphocytes, but interestingly revealed a low positivity (<8%) for CD34, CD45 and CD106 [27]. This data suggests that the infiltration of immune cells occurs to varying degrees in patients with different joint injuries and is also likely to vary at different stages of the disease. The SF cells described in the ligament injury study were evaluated at an earlier stage in disease progression than in our patients, which could indicate that the establishment of an inflammatory state within the joint is progressive and more pronounced in our patients, when considering the higher relative levels of CD106 and CD34 observed in our study.

A number of the SF samples investigated in the present study did not contain cells that met the recommended criteria for MSCs [28] following culture expansion, due to the positivity of CD73 and CD90 being below 95%, which supports the theory that there is a mixed population of cells present in the SF of these patients. Furthermore, these results corroborate observations by another group where human SF-MSCs, obtained before and after ligament surgery, did not fully meet the immunoprofile criteria for MSCs [27,28]. The cause of the focal defects is unknown in our cohort of patients, which makes it difficult to establish a link between initial trauma and cell infiltration into the joint space. Attempts have been made to understand this process in animal injury models of OA [[29], [30], [31], [32]], but this requires further research in humans for the improved understanding of attempted joint repair by endogenous stem cell niches.

Previous research has revealed the presence of cells with chondrogenic ability in the SF of end-stage OA patients [10], however, only a few studies have explored these cells following cartilage injury or in the early stages of OA [9]. In the current study, surface markers and genes known to be indicative of chondrogenic potency in chondrocytes, bone marrow MSCs and adipose cells [14], were used to assess SF cells. In comparison, the expression levels of the chondrogenic genes tested for in the SF cells (for Sox9, collagen type II, aggrecan and frizzled-related protein 1) were unsurprisingly lower than what we observed in articular chondrocytes, whereas the hypertrophic genes (collagen type X and activin receptor-like kinase 1) were expressed at similar levels in SF cells and chondrocytes [14]. None of the markers tested correlated with the chondrogenic ability of SF cells until the statistical outlier (patient 4) was removed, after which CD90 and CD105 immunopositivity negatively correlated with GAG/DNA production and *COL2A1* expression positively correlated with GAG/DNA. It is not too surprising that higher *COL2A1* expression levels relate to increased pellet GAG content as both are established indicators of chondrogenic differentiation. However, the finding that CD90 and CD105 immunopositivity negatively correlates with GAG content is interesting as it has been suggested that these markers relate to enhanced chondrogenic potential [33,34], although others have disputed this [35]. Patient 4 had no unusual clinical or biological features to explain the superior chondrogenic ability of their cells, other than, perhaps, being the youngest patient of the cohort (19 years old). It is noteworthy that the quantity of GAG produced by SF cells in the present study was lower than we reported for chondrocytes and infrapatellar fat pad derived MSCs, but similar to bone marrow and subcutaneous fat derived MSCs [14]. The heterogeneous metachromatic staining of the chondrogenic pellets suggests that there may be a subpopulation of SF cells (MSCs, chondrocytes or chondroprogenitor cells) with enhanced chondrogenic ability.

It is believed that MSCs may be recruited from various joint tissues such as the synovium, bone marrow, fat pads, cartilage and ligaments when injury or damage occurs within the joint [27,29]. MSCs are also known to be present in peripheral blood [36] and as such it is possible that blood derived MSCs could be found in the joint from intra-articular bleeding. The SF from joints with end-stage OA contains more MSCs than healthy joints [9] and it could be hypothesised that the patients in the present study did not have sufficient joint degeneration to trigger the recruitment of numerous MSCs into the SF. It has been suggested that chemoattractant molecules released from damaged ligaments could be responsible for the increase in SF-MSCs noted after ligament surgery [27]. The patients included in our study had no indication of ligament damage in addition to their focal cartilage defects and other studies have shown that damaged cartilage releases chemokines that recruit immune cells [[37], [38], [39]], but it remains unclear whether MSC infiltration is also initiated in this process.

To investigate the various subsets of CD14^+^ macrophages present in the SF of patients receiving treatment for cartilage injury, we assessed the positivity of known M1 (CD80 and CD86) and M2 (CD163 and CD206) markers. To our knowledge, our results are the first to show the co-existence of cells with both M1 and M2 markers in SF of early OA patients. The markers CD80 and CD86 are co-stimulatory factors involved in antigen presentation and T cell activation and are associated with a pro-inflammatory phenotype [17,18], while CD163 (scavenger receptor) and CD206 (mannose receptor) are associated with an anti-inflammatory and wound healing phenotype [15,16], The maturation of macrophages from circulatory monocytes is mediated by some soluble factors including tumour necrosis factor-alpha (TNF-α), interleukin-4 (IL-4), interleukin-10 (IL-10) and interleukin-13 (IL-13); TNF-α would polarise the cells to an M1 phenotype, while IL-4, IL-10 and IL-13 would polarise the cells to an M2 phenotype [40]. We have shown that IL-10 and IL-13 are detectable in the SF of patients with cartilage defects [41,42], while the presence of TNF-α and other pro-inflammatory molecules in OA knee joints has been widely reported [43]. Intra-articular bleeding could explain the SF macrophage population. In addition, the presence of macrophage subsets has been confirmed in the synovium and infrapatellar fat pad in end-stage OA joints [44,45], which could represent a local source. We found that some macrophages co-express both M1 and M2 markers, which adds to the growing body of literature challenging the dichotomous model of macrophage polarisation being either pro- or anti-inflammatory and indicating that these macrophages can perform both functions [46,47]. Our results complement the findings of a recent study investigating SF cells from end-stage OA joints, which similarly noted the presence of macrophages with overlapping pro- and anti-inflammatory phenotypes [48]. Assessing the positivity of the various macrophage subsets in matched samples at baseline and 2–3 weeks after cartilage harvest in ACI has allowed for a rare snapshot of the cellular response of SF to acute injury and in the presence of established focal defects. We have previously shown that there is a detectable response to stage 1 surgery in the SF proteome and for many patients, acute phase response signalling proteins are significantly upregulated [49]. There is a likely role for the macrophages identified in this study in this acute phase response, which warrants further study in a larger sample cohort.

The co-existence of immune cells and MSCs in the SF of patients with cartilage injuries and at an early stage of OA development could have implications for the wound healing and regenerative capacity of a joint. We previously demonstrated the negative effects of conditioned media from M1 polarised macrophages on the chondrogenic ability of BM-MSCs, with a similar inhibitory effect being observed with conditioned media from OA synovium and infrapatellar fatpad explant cultures [44,50]. This collective body of work, including the present study, shows that there could be interplay between macrophages (and other innate immune cells) and MSCs, possibly to orchestrate an endogenous repair. Longitudinal *in vivo* studies would be required to disentangle these processes of attempted repair, but also to understand when and why this repair often fails. The early stages of joint disease offer a window of opportunity for treatment, but unravelling the phenotypes of SF cells, as we have in this study, needs to be further investigated.

This investigation has some limitations, the main one being the relatively small sample size. We also acknowledge that other confounding factors, such as the level of systemic inflammation and the surgery itself, could have an influence on the phenotype of SF cells. Furthermore, our study only gives a snapshot of the cellular phenotypes at a given time of the disease state, and is unlikely to represent the entire spectrum of different cell states at the various phases of OA progression. Our results further highlight the disconnect between the expression of key chondrogenic markers and the actual chondrogenic output of MSCs. Comparing our data to SF cells from healthy joints would have been ideal, however we did not have access to such samples. The phenotype of SF cells only represents a part of the “bigger picture” of early OA and other joint tissues, such as the damaged cartilage and inflamed synovium, would also need to be investigated and compared to the SF cells for a more comprehensive characterisation.

In conclusion, the results presented here revealed that the SF of patients with focal cartilage defects contains a mixed population of adherent cells that likely include monocytes/macrophages, endothelial cells, synoviocytes, chondrocytes and MSCs. The macrophage subsets in the SF from this group of patients, which express both pro- and anti-inflammatory markers, shows the presence of both damaging and reparative phenotypes, indicative of the cocktail of different signals present in the joint. Further research into the phenotype of macrophages within the joint could shed new light on the pathogenesis of OA. Follow-on studies should be geared towards creating a more detailed timeline of the cellular mechanisms involved following cartilage injury and understanding more fully the ‘stage specific’ roles of the various cellular subsets identified.

## Declaration of Competing Interest

The authors have no conflict of interest to declare.

## Figures and Tables

**FigS1:**
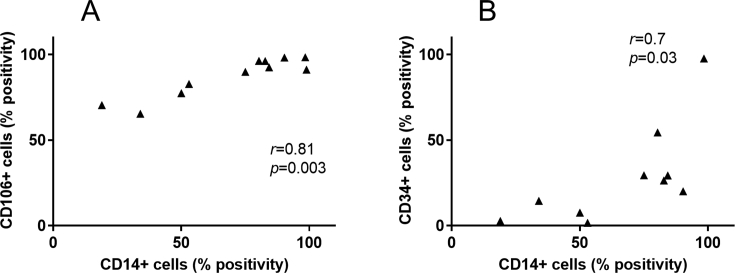


**FigS2:**